# Research on the Method of Reducing Dynamic Cutting Force in Aspheric Machining

**DOI:** 10.3390/mi14050960

**Published:** 2023-04-28

**Authors:** Guilin Zhuang, Hanzhong Liu, Wenjun Zong

**Affiliations:** Center for Precision Engineering, Harbin Institute of Technology, Harbin 150001, China; 20b908133@stu.hit.edu.cn

**Keywords:** spherical/aspheric surface, dynamic cutting forces, workpiece shape, interpolating algorithm, tool tip radius

## Abstract

With the rapid development of photoelectric communication and other fields, the demand for high-precision aspheric mirrors has been increasing. Predicting dynamic cutting forces is vital in selecting machining parameters and also affects the surface quality of the machined surface. This study comprehensively considers the effects of different cutting parameters and workpiece shape parameters on dynamic cutting force. The actual width of cut, depth of cut, and shear angle are modelled while considering the effects of vibration. A dynamic cutting-force model considering the aforementioned factors is then established. Using experimental results, the model accurately predicts the average value of dynamic cutting force under different parameters and the range of fluctuation of dynamic cutting force, with a controlled relative error of about 15%. The influence of workpiece shape and workpiece radial size on dynamic cutting force is also considered. The experimental results show that the greater the surface slope, the more dramatic the dynamic cutting force fluctuations. This lays the foundation for subsequent writing on vibration suppression interpolation algorithms. The influence of the radius of the tool tip on dynamic cutting forces leads to the conclusion that to achieve the goal of reducing the fluctuation of cutting forces, diamond tools with different parameters should be selected for different feed rates. Finally, a new interpolation-point planning algorithm is used to optimize the position of interpolation points in the machining process. This proves the reliability and practicability of the optimization algorithm. The results of this study are of great significance to the processing of high-reflectivity spherical/aspheric surfaces.

## 1. Introduction

In recent years, due to rapid developments in micromachining technology, the requirement for workpiece-surface machining accuracy has increased significantly. Vibration during machining is one of the most significant factors that affect surface roughness. The vibration of the machine tool system can be categorized into three types: the dynamics of the machine tool’s moving parts, the cutting dynamics, and chatter. The latter can be further classified into material-induced vibration, tool–workpiece relative vibration, and chatter. A systematic study of material-induced cutting vibrations was conducted by Lee et al. [1,2,3,4,5], which indicated that shear strength and shear angle vary with crystal orientation, leading to unstable cutting during the cutting process. Wang et al. [6,7,8,9] researched tool tip vibration and discovered two vibration frequencies with high frequency and low amplitudes between 13 kHz and 14 kHz. The analysis of the cutting-force signal has led to the conclusion that the two vibration frequencies are due to the impact of the tool–chip contact surface caused by the elastic recovery of the workpiece surface. The introduction of the CPR index permitted quantification of these two high-frequency-vibration bimodal peaks. The simulation demonstrates that the CPR index and surface roughness are highly correlated and concludes that the main factor influencing surface roughness is tool tip vibration.

Cutting vibrations induce a change in the actual depth and width of the tool’s cut, leading to fluctuations in cutting forces that are referred to as dynamic cutting forces. Currently, there exist numerous research findings on dynamic cutting forces. Wojciechwski et al. [10] considered the process of rolling and elastic recovery of workpiece materials by cutting edge in micro-milling and established a micro-milling force model considering the accumulation of chip thickness, the tool vibration of micro-milling cutter and the flexibility of milling cutter itself. Ortega et al. [11] established a new transient undeformed chip thickness model. The experimental results show that the relative error of the model is stable at about 2%, which lays the foundation for an accurate milling force model. Because the contact area between the tool and the workpiece depends on the deflection angle between the tool and the workpiece, Utsumi et al. [12] considered the effect of the milling tool position on the milling forces, and using point cloud techniques, developed a model of the milling forces considering the attitude of the tool. Zhang et al. [13] puts forward a comprehensive evaluation method based on long short-term memory (LSTM) and particle filter (PF) algorithm to monitor the random wear of cutting tools in real time. A milling force model considering random tool wear, tool runout and actual tool movement is established. Compared with the traditional milling force model, the accuracy of this model is improved by 3.4%. Behera et al. [14] developed a cutting force model considering the tool–work contact area under MQL lubrication conditions, which integrated the effects of vibration, chip thickness and tool–chip contact length on cutting forces.

To consider cutting forces in the presence of vibrations is essentially to take into account the impact of vibrations on the thickness and width of the cut as well as the actual shear angle. Zhang et al. [15] integrated the effect of tool run out on the actual chip thickness and rake angle, and developed a milling force model that considered the effect of ploughing on the flank tool face. In the process of vibration-assisted drilling of titanium alloy, Li et al. [16] considered the influence of vibration-assisted process on chip generation. Because the chip shape has a great influence on the drilling force, the drilling force model is modified according to the chip shape. It is concluded that the auxiliary vibration process reduces the contact length of chips by 26.6–43.9%. Regarding the study of the vibration process on the instantaneous undeformed chip thickness, Jing et al. [17] developed a model for the instantaneous chip thickness considering tool runout and modified the cutting force coefficient. Lin et al. [18] combined the oblique elliptical cutting (EVC) process with a comprehensive analysis of the variation of parameters such as chip thickness and shear angle during EVC cutting and established a cutting force model under the EVC process, which can predict the dynamic cutting force well.

Dynamic cutting force plays a critical role in determining the service life of cutting tools, the cutting temperature, and the stability of machine tools. Although significant research has been conducted on dynamic milling forces in flat turning in recent years, there has been relatively little attention on dynamic cutting forces during spherical/aspheric surface turning. In this paper, dynamic cutting forces during spherical/aspherical machining are modelled; actual cutting width and depth are modelled taking into account cutting vibrations. For the first time, we consider the influence of different interpolation algorithms on the actual cutting depth and the influence of the speed change of adjacent interpolation points on the cutting force. Through experiments, we obtained the influence of cutting parameters, tool parameters and workpiece shape on dynamic cutting force. The first-time discussion of the impact of workpiece shape on dynamic cutting force is presented. The greater the curvature of the workpiece surface, the greater the change in dynamic cutting force. Additionally, the effect of using different interpolation algorithms on dynamic cutting forces is investigated. Based on theoretical models and experimental results, it is apparent that different interpolation algorithms significantly affect dynamic cutting forces, and the effectiveness of selecting a suitable interpolation algorithm to reduce kinetic fluctuations is assessed.

## 2. Dynamic Cutting Force Modelling

**Figure 1:**
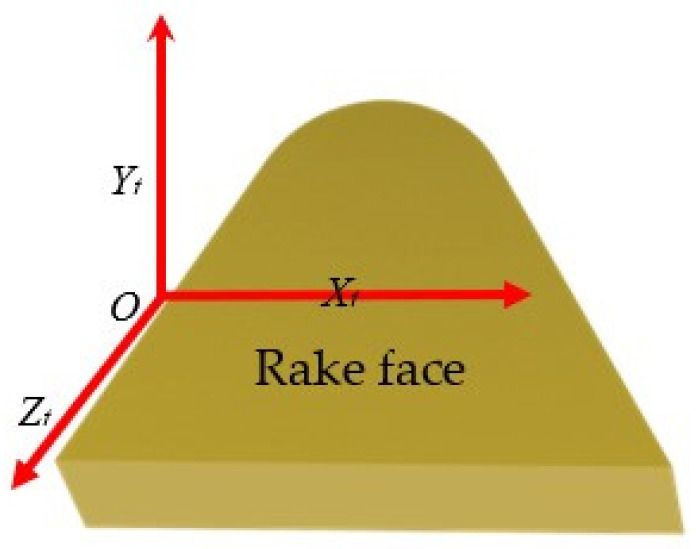
Tool coordinate system.

Before establishing the dynamic cutting force of the tool during vibration, the coordinate system of the tool tip point should be established as follows: *O* − *X_t_Y_t_Z_t_* (As shown in Figure 1). Firstly, the cutting forces on the tool are modelled (taking into account the different depths of cut, the contact between the rake face of the tool and the chip or not). Then, the influence of cutting parameters, such as cutting depth and feed rate, on the amplitude and frequency of nonlinear vibration is studied.

According to Zhang’s article [19], we divided the vibration between the tool–workpiece into two directions, the *Z*-direction and the *Y*-direction

(1) $$\left\{ \begin{array}{l} m{\ddot{Z}}_{t}+c_{z}{\dot{Z}}_{t}+k_{z}Z_{t}=F_{z}\\ m{\ddot{Y}}_{t}+c_{y}{\dot{Y}}_{t}+k_{y}Y_{t}=F_{y} \end{array}\right.$$

where *m* is the respective effective mass, *c_n_* (*n* = *z,y*) is the respective damping coefficients, *k_n_* (*n* = *z,y*) is the respective stiffness, and *F_n_* (*n* = *z,y*) is the respective dynamic cutting force.

### 2.1. Modelling of Cutting Stress without Vibration

**Figure 2:**
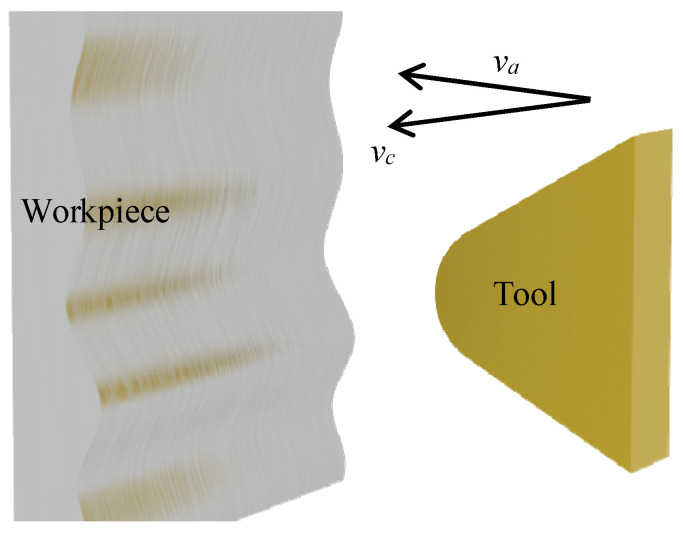
Schematic diagram of tool radial feeding velocity.

According to relevant research, the change of cutting speed direction caused by vibrations and the radius of blunt circle of cutting edge will lead to the change of the actual cutting rake angle. Diamond tools in the cutting process due to the influence of vibration, the direction of its cutting speed changes at all times, and the angle of change by the following formula can be obtained using

(2) $$\alpha_{v}=\arctan\left(\frac{v_{a}}{v_{c}}\right)$$

where $v_{a}$ is the radial feed speed of the tool as shown in Figure 2, and $v_{c}$ is the actual cutting speed of the tool.

The actual rake angle $\alpha_{r}$ is

(3) $$\alpha_{r}=\alpha +\alpha_{v}$$

where α is the nominal rake angle of the tool.

The nominal rake angle takes the value 0° so that the actual rake angle $\alpha_{r}$ = $\alpha_{v}$.

The expression for the actual shear angle is

(4) $$\phi =\phi_{0}+\phi_{s}$$

where $\phi_{s}$ is the shear angle determined by the original surface of the workpiece [20], and $\phi_{0}$ is the nominal shear angle.

As the shear angle is determined, accordingly, the position of the shear face can be determined. The shear strain on the shear face is influenced by the magnitude of each component of the slip velocity of the shear face so that the expression for the shear strain on the shear surface is obtained as

(5) $$\gamma_{s}=\frac{1}{2}\frac{v_{s}}{v_{⊥}}$$

where $v_{s}$ is the shear surface movement velocity, whose direction is parallel to the shear surface [19], and the expression is

(6) $$v_{s}=\frac{v_{c}\cos\alpha_{r}}{\cos(\phi -\alpha_{r})}$$

where $v_{⊥}$ is the velocity perpendicular to the shear plane and expressed as

(7) $$v_{⊥}=v_{c}\cdot \sin\phi$$

When substituting the above two equations into Equation (8), the shear strain $\gamma_{s}$ is

(8) $$\gamma_{s}=\frac{\cos(\alpha_{r})}{2\cdot \sin(\phi )\cdot \cos(\phi -\alpha_{r})}$$

Using Oxley’s theory [21], the shear strain rate in the shear zone can be obtained as

(9) $${\dot{\gamma }}_{s}=\frac{C_{ox}v_{s}}{l_{s}}$$

where $C_{ox}$ is the Oxley factor, $l_{s}$ is the shear band length, which is expressed as

(10) $$l_{s}=\frac{t_{u}}{\sin\phi }$$

Drawing on the experience of previous studies, the *J-C* intrinsic structure equation is used in this paper to describe the intrinsic structure of the material. Combined with the related conclusions in Section 2.2, the energy balance inside the shear plane is approximately [21]

(11) $$\rho \cdot c\cdot \left(T_{s}-T_{0}\right)=(1-R)\tau_{s}\gamma_{s}$$

where *c* is the specific heat capacity of the workpiece material, ρ is the density of the workpiece material, $T_{s}$ is the actual temperature of the shear surface, $T_{0}$ is the ambient temperature (set to 20 °C in this paper), and *R* is the proportion of heat flow from the first deformation zone to the second deformation zone.

Therefore, by combining the above formula with the J-C constitutive equation, the expression of shear stress is obtained as follows:

(12) $$\tau_{s}=\frac{m\cdot \rho \cdot c\cdot (T_{m}-T_{0})}{\sqrt{3}\rho \cdot c\cdot (T_{m}-T_{0})+m\cdot \left(1-R\right)\gamma_{s}\Gamma_{JC}}\cdot \Gamma_{JC}$$

where $\Gamma_{JC}$ is the *J-C* instanton equation, *m* is the constant characterizing the material and *T_m_* is the melting point of the workpiece material.

From the above equation for the shear stress in the shear zone, it can be obtained that the shear stress is no longer a constant value. Therefore, along the shear slip, line the individual stresses will have the following expressions

(13) $$\left\{ \begin{array}{l} \frac{\partial p}{\partial s_{1}}+2\tau_{s}\frac{\partial \psi }{\partial s_{1}}-\frac{\partial \tau_{s}}{\partial s_{2}}=0\\ \frac{\partial p}{\partial s_{2}}-2\tau_{s}\frac{\partial \psi }{\partial s_{2}}-\frac{\partial \tau_{s}}{\partial s_{1}}=0 \end{array}\right.$$

where *s*_1_ and *s*_2_ are the specified distances along the slip line fields I and II, *p* is the hydrostatic pressure along the slip line, and ψ is the angle between the slip line along the counterclockwise direction and class I in the specified coordinate system.

It was found [22] that when the loading history is negligible, shear stress is available as a function of shear strain, shear strain rate and temperature as shown in the following expressions (with the direction of the shear plane as the *w*-axis and the direction perpendicular to the shear plane as the *u*-axis)

(14) $$\frac{\partial \sigma }{\partial u}=\frac{\partial \tau_{s}}{\partial w}=\frac{\partial \tau_{s}}{\partial \gamma_{s}}\frac{\partial \gamma_{s}}{\partial t}\frac{\partial t}{\partial w}+\frac{\partial {\dot{\gamma }}_{s}}{\partial w}\frac{\partial \tau_{s}}{\partial {\dot{\gamma }}_{s}}+\frac{\partial \tau_{s}}{\partial T_{s}}\frac{\partial T_{s}}{\partial w}$$

As the shear strain rate is generally assumed to be at the maximum on the shear surface during the study [22], the second term to the right of the above equation is approximated as zero, and considering the small range of temperature gradient variation on the shear face, the third term to the right of the above equation is also approximated as zero.

From Hencky’s stress theorem we have

(15) $$\sigma_{s1}-\sigma_{s2}=l_{s}\frac{\Delta k}{\Delta s}=l_{s}\frac{\partial \tau_{s}}{\partial w}$$

where $\sigma_{s1}$ and $\sigma_{s2}$ are the stress values at the two boundary endpoints of the shear surface slip-line, respectively.

Combining the two equations above, we get

(16) $$\sigma_{s1}-\sigma_{s2}=l_{s}\frac{\partial \tau_{s}}{\partial w}=l_{s}\frac{\partial \tau_{s}}{\partial \gamma_{s}}\frac{\partial \gamma_{s}}{\partial t}\frac{\partial t}{\partial w}$$

The relationship between the stresses at the two endpoints along the shear zone can also be obtained from $\frac{\partial \gamma_{s}}{\partial t}={\dot{\gamma }}_{s}$ and $\frac{\partial t}{\partial w}=\frac{1}{v_{⊥}}$.

(17) $$\sigma_{s1}-\sigma_{s2}=\frac{t_{u}}{\sin(\phi )}\frac{{\dot{\gamma }}_{s}}{v_{⊥}}\frac{\partial \tau_{s}}{\partial \gamma_{s}}$$

where, from the *J-C* instanton equation we get

(18) $$\frac{\partial \tau_{s}}{\partial \gamma_{s}}=\tau_{S}\cdot \left[\frac{Bn}{\gamma_{s}}{\left(\frac{\gamma_{s}}{\sqrt{3}}\right)}^{m}\right]/\left[A+B{\left(\frac{\gamma_{s}}{\sqrt{3}}\right)}^{m}\right]$$

Therefore, by combining the above equations, we can obtain an expression for the relationship between the stresses at the two endpoints of the shear face as

(19) $$\sigma_{s1}-\sigma_{s2}=2\tau_{s}\cdot m\cdot \frac{B\cdot C_{ox}\cdot \gamma_{s}^{m}}{{\sqrt{3}}^{m}\cdot A+B\cdot \gamma_{s}}$$

By the theory of Oxley and other scholars [22], we can get the $\sigma_{s1}$ on the shear face as

(20) $$\sigma_{s1}=\tau_{s}\cdot \left[1+2\left(\frac{\pi }{4}-\phi \right)\right]$$

By substituting Equation (20), we can get the maximum stress value on the rake face. According to existing research [23], the expression for the chip contact length on the rake face can be obtained as

(21) $$l_{con}=\frac{2\cdot t_{u}}{\tan(\vartheta )}$$

Based on the known contact length and the maximum positive contact stress on the rake face obtained above, the distribution of the stress on the rake face with the distance *t_s_* from the tool tip to the rake face can be obtained as

(22) $$\sigma (s)=\sigma_{s2}\left[1-{\left(\frac{t_{s}}{l_{con}}\right)}^{a}\right]$$

where *a* is the power index, which is set to a constant in this paper to simplify the model, and *l_con_* is the tool–chip contact length.

For the friction distribution on the rake face, the tool–chip friction coefficient μ is assumed to remain constant along the rake face. It is known that this can be divided into two parts: the bonding zone and the slipping zone.

When it reaches a certain distance from the tip of the tool, there is a sliding phenomenon between the tool and the chip, and then sliding friction will happen. Therefore, the friction stress on the whole rake face can be expressed as

(23) $$\tau_{f}=\left\{ \begin{array}{l} \tau_{st},\begin{array}{cc} & s\in \left[0,l_{d}\right] \end{array}\\ \mu \cdot \sigma (s),s\in \left[l_{d},l_{con}\right] \end{array}\right.$$

From previous studies [24], it is generally taken as ld=34lcon. The flow stress $\tau_{st}$ can be expressed as

(24) $$\tau_{st}=\mu \cdot \sigma_{s2}(1-2^{-a})$$

Contact between the flank face of the tool and the workpiece happens and ploughing happens. When plastic deformation happens, ploughing and friction forces are generated, and the plastic deformation force (ploughing force) is expressed as [24]

(25) $$\left\{ \begin{array}{l} \tau =\tau_{s}\left(1-\frac{x}{s}\right)\sin\left(\beta_{0}\right)\\ \sigma =\frac{\tau }{\mu } \end{array}\right.$$

where τ and σ are the shear and normal stresses on the contact interface between the flank face and the machined surface, respectively, $\tau_{s}$ is the shear stress on the shear plane ahead of the active cutting edge, *s* is the material spring back, $\beta_{0}$ is the tool flank angle, and *x* is the distance from the tool tip to a concerned point at the tool–workpiece contact interface.

### 2.2. Effect of Different Interpolation Algorithms on Cutting Forces

In the process of machining aspheric surfaces, there are two common methods for planning tool paths, namely, equal feed cutting and equal residual height cutting. The following two methods are used to model the cutting depth affected by vibrations.

#### 2.2.1. Equal Feed Cutting

As the name implies, equal feed cutting means that the feed values between two adjacent cutting paths are equal [25]. This path planning method is commonly used at present. Because the slopes of aspheric surfaces at the interpolation points on meridian are not equal, the cutting depth will change with the slope of meridian in constant feed cutting; the greater the slope, the greater the depth of cut.

**Figure 3:**
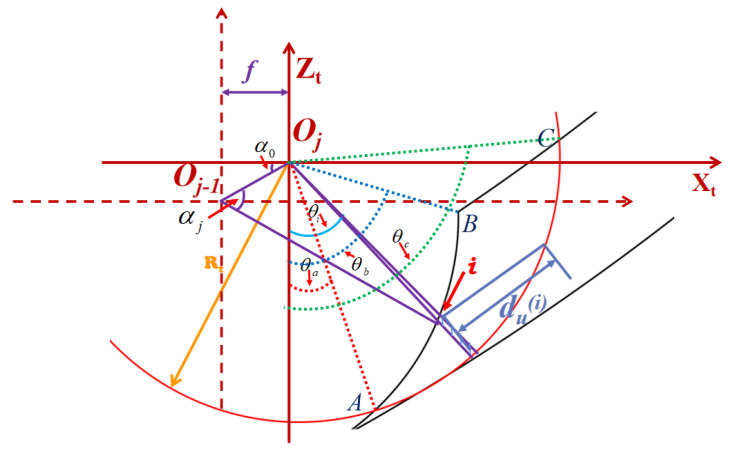
Schematic diagram of the depth of cut during aspheric turning.

The schematic diagram of the depth of cut of the tool under the two adjacent paths is shown in Figure 3.

The depth of cut produced by two adjacent tool paths is shown schematically in Figure 2. In this figure, A and B are the two end points of the cutting width. *O_j_* is the original point of the tool coordinate system *X_t_* − *O_j_* − *Z_t_* in response to the *j*th tool path. *O_j_*_−1_ is the original point of the tool coordinate system *X_t_* − *O_j_*_−1_ − *Z_t_* with respect to the (*j* − 1) th tool path. *i* is an arbitrary point on the cutting width. *f* is the feed rate, and *t_u_* is the cutting width. According to the geometrical relationship as described in this figure, the angle $\alpha_{i}$ is calculated as

(26) $$\alpha_{j}=\frac{\pi }{2}+\alpha_{0}-\theta_{i}-\arcsin\left[\frac{d}{R_{T}}\cdot \cos\left(\theta_{i}-\alpha_{0}\right)\right]$$

where *d* is the length of *O_j_*_−1_*O_j_* and its equation is $d=\sqrt{f^{2}+{\left(z_{j-1}-z_{j}\right)}^{2}}$, *z_j_*_−1_ and *z_j_* are the ordinate of point *O_j_*_−1_ and point *O_j_*, respectively, $\alpha_{0}$ is the angle between the line *O_j_*_−1_*O_j_* and the *X_t_*-axis, and $\theta_{i}$ is the relative angle between the *i*th cutting piece and the *Z_t_*-axis.

The expression for the depth of cut is

(27) $$t_{u}^{\left(i\right)}=\left\{ \begin{array}{l} R_{T}-R_{T}\frac{\cos\left(\alpha_{0}-\theta_{i}-\arcsin\left[\frac{d}{R_{T}}\cdot \cos\left(\theta_{i}-\alpha_{0}\right)\right]\right)}{\cos\left(\theta_{i}-\alpha_{0}\right)}\theta_{i}\in \left[\theta_{a},\theta_{b}\right)\\ R_{T}-\left|\frac{z_{T}^{\left(i\right)}}{\sin\left(90^{\circ }-\theta_{i}\right)}\right|\theta_{i}\in \left(\theta_{b},\theta_{c}\right] \end{array}\right.$$

where $z_{T}^{\left(i\right)}$ is the *ZZ* coordinate of point *I*, and $\theta_{a}$, $\theta_{b}$ and $\theta_{c}$ are the included angles between the lines *O_j_A*, *O_j_B* and *O_j_C* and the *Z_t_*-axis, respectively, as marked in Figure 3.

#### 2.2.2. Equal-Residual-Height Cutting

In the equal-residual-height cutting mode [26], it should ensure that the residual heights of any two adjacent cutting paths are equal, which requires the feed rate to dynamically vary with the surface slope of workpiece, supposing that the expression of the workpiece surface is *g*(ρ,θ).

According to the definition of equal-residual-height interpolation algorithm, the interval *L_j_*between two adjacent tool paths, *N_j_* and *N_j+_*_1_, can be expressed as

(28) $$L_{j}=\sqrt{\frac{8R_{T}\varepsilon_{t}}{1-R_{T}\zeta_{j}}}$$

where $\varepsilon_{t}$ is the residual error as required to control the residual height in planning tool paths, and $\zeta_{j}$ is the surface curvature in response to the *j*th tool path.

In addition, the surface slope *dz_j_* of the *j*th tool path can be calculated as

(29) $$dz_{j}=\min\left\{{\frac{\partial g}{\partial \rho }|}_{\rho =\rho_{j}},\forall \theta \in \left[0^{\circ },360^{\circ }\right)\right\}$$

The feed rate *f’* in an equal residual height cutting mode can be formulated as

(30) $$f^{\prime }=\frac{L_{j}}{\sqrt{1+{\left(dz_{j}\right)}^{2}}}$$

By substituting Equation (30) into Equation (27), the actual depth of cut and cutting width for the equal-residual-height cutting can be determined.

By substituting into Section 2.2, we can get the dynamic cutting forces of different interpolation algorithms.

### 2.3. Cutting Force Modeling

Therefore, according to the friction behavior, the cutting forces *F_z_*_1_ and *F_x_*_1_ on the rake face is modelled as

(31) $$F_{z1}=\int_{\theta_{A}}^{\theta_{C}}\left(\int_{0}^{l_{d}}\tau_{st}dl+\int_{l_{d}}^{l_{con}}\mu \cdot \sigma \left(t_{s}\right)dl\right)\cdot \cos\left(\theta_{i}\right)d\theta_{i}$$

where *F_z_*_1_ is the cutting forces along the *Z*-directions, respectively.

**Figure 4:**
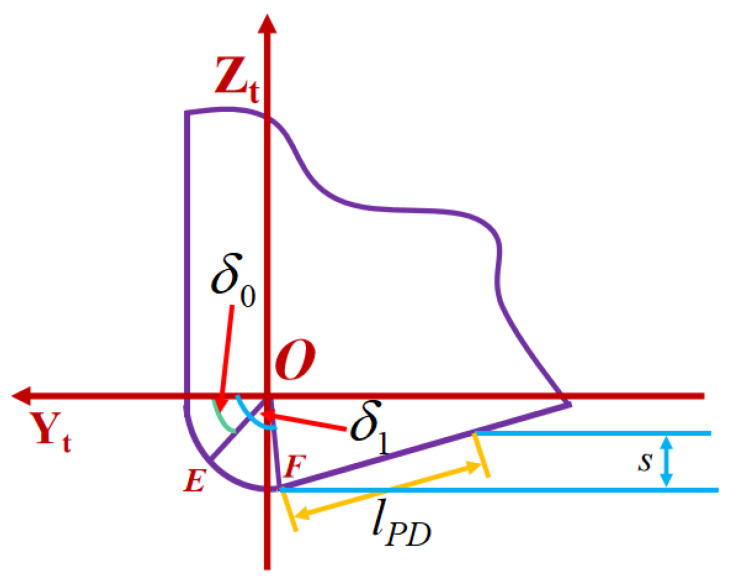
Cutting edge angle range.

According to the stress distribution predicted by Equation (25), the three-dimensional cutting forces on the flank face and cutting edge can be formulated as

(32) $$\left\{ \begin{array}{l} F_{z2}=\int_{\theta_{A}}^{\theta_{C}}\int_{0}^{l_{PD}}\left(\tau \cos\left(\theta_{i}\right)-\sigma \sin\left(\theta_{i}\right)\right)R_{T}dxd\theta_{i}+\int_{\theta_{A}}^{\theta_{C}}\int_{\delta_{0}}^{\delta_{1}}\left(\tau \sin\left(\delta \right)+\sigma \cos\left(\delta \right)\right)R_{T}r_{n}d\delta d\theta_{i}\\ F_{y2}=\int_{\theta_{A}}^{\theta_{C}}\int_{0}^{l_{PD}}\left(\tau \sin\left(\theta_{i}\right)+\sigma \cos\left(\theta_{i}\right)\right)R_{T}\cos\left(\theta_{i}\right)dxd\theta_{i}+\int_{\theta_{A}}^{\theta_{C}}\int_{\delta_{0}}^{\delta_{1}}\left(-\tau \cos\left(\delta \right)+\sigma \sin\left(\delta \right)\right)R_{T}\cos\left(\theta_{i}\right)r_{n}d\delta d\theta_{i} \end{array}\right.$$

where *R_T_* is the tool nose radius, the cutting edge angle range satisfies $\delta_{0}<\delta <\delta_{1}$; $\delta_{0}$ is the angle between the chip separation line *OE* and *Y*-axis; $\delta_{1}$ is the angle between the boundary line *OF* the cutting edge of the cutter and *Y*-axis as shown in Figure 4; *l_PD_* is the total contact length between the flank face and the machined surface, and $l_{PD}=\frac{s}{\sin\left(\beta_{0}\right)}$; *F_y_*_2_, *F_z_*_2_ are the two-dimensional cutting forces on the flank face; and *r_n_* is the tool blunt radius.

When machining aspheric surfaces, the interpolation algorithm specifies that the speed of each interpolation point moves along the tangential direction of the interpolation point. Each point on the surface does not have the same tangential direction, and the change in velocity direction generates a corresponding acceleration that acts on the tool tip, causing it to be subjected to the corresponding external forces.

The feed velocity per revolution is calculated as

(33) $${\vec{v}}_{f}=\frac{f_{s}\cdot n}{60}$$

The expression for the velocity at a given interpolation point ${\vec{v}}_{i}$ is

(34) $${\vec{v}}_{i}=\frac{{\vec{v}}_{f}}{\cos\left(\beta_{i}\right)}$$

where $\beta_{i}$ is the angle between the tangent to the surface and the axis of rotation, and the expression is $\beta_{i}=\frac{1}{\zeta_{i}}$.

$\zeta_{i}$ is the curvature at point *i*, and its expression is

(35) $$\zeta_{i}=\min\left\{{\frac{\partial^{2}g/\partial^{2}\rho }{{\left[1+\partial g/\partial \rho \right]}^{\frac{3}{2}}}|}_{\rho =\rho_{i}},\forall \theta \in \left[0^{\circ },360^{\circ }\right)\right\}$$

The expression for the velocity ${\vec{v}}_{i+1}$ at its neighboring interpolation points is

(36) $${\vec{v}}_{i+1}=\frac{{\vec{v}}_{f}}{\cos\left(\beta_{i+1}\right)}$$

Then, the difference in velocity Δv between two adjacent interpolation points is

(37) $$\Delta v=\sqrt{{\left(\vec{v_{i}}\right)}^{2}+{\left(\vec{v_{i+1}}\right)}^{2}-2\left|\vec{v_{i}}\right|\cdot \left|\vec{v_{i+1}}\right|\cdot \cos\left(\gamma_{i}\right)}$$

where $\gamma_{i}$ is the angle between the two interpolation points along the direction perpendicular to the tangent to the radius of the surface, $\gamma_{i}=\beta_{i+1}-\beta_{i}$.

Thus, the acceleration *a_i_* between two adjacent interpolation points is obtained as

(38) $$a_{i}=\frac{\Delta v}{\Delta t}$$

In turn, we can obtain an expression for the external force at the tip of the tool due to the velocity difference

(39) $$F_{i}=m\cdot a_{i}$$

where *m* is the respective effective mass.

Therefore, to sum up, we get the cutting forces in the two directions as

(40) $$\left\{ \begin{array}{l} F_{y}=F_{y2}\\ F_{z}=F_{z1}+F_{z2}+F_{i}\cdot \sin\left(\gamma_{i}\right) \end{array}\right.$$

### 2.4. Influence on Cutting Stress during Tool-Workpiece along Z-Vibration and Y-Vibration

When the tool tip produces the translational vibration along the *Z*-axis, the direction of the friction force on tool rake face is related to the direction of the relative velocity Δv between the tool and the chip. In this case, the frictional stress on the rake face can be written as

(41) $$\tau_{f}^{\prime }=\left\{ \begin{array}{l} \tau_{st},\begin{array}{cc} & s\in \left[0,l_{d}\right] \end{array}\\ \mu \cdot \sigma (s)\cdot \mathrm{sgn}\left(\Delta v\right),s\in \left[l_{d},l_{con}\right] \end{array}\right.$$

In addition, once the tool–workpiece vibration takes place along the *Z*-direction and *X*-direction, the actual depth of cut *t_u_* and cutting width *d_u_* will change, and the variations $t_{u}^{\prime }$ and $d_{u}^{\prime }$ can be calculated as

(42) $$\left\{ \begin{array}{l} t_{u}^{\prime }=t_{u}-\mathrm{sgn}\left(v_{a}-{\dot{Z}}_{t}\right)\cdot Z_{t}\cdot \cos\left[\arctan\left(dz_{j}\right)\right]\\ d_{u}^{\prime }=d_{u}-\mathrm{sgn}\left(v_{a}-{\dot{Z}}_{t}\right)\cdot Z_{t}\cdot \sin\left[\arctan\left(dz_{j}\right)\right] \end{array}\right.$$

where *v_a_* is the tool feed speed along the *Z*-direction, *dz_j_* is the surface slope corresponding to the *j*th tool path, which will be discussed in the following section, *X_t_* is the vibration induced displacements of the tool and workpiece along the *X*-direction, and *Z_t_* is the vibration induced displacements of the tool and workpiece along the *Z*-direction.

Due to the vibration along the *Y*-direction, the duty cycle *DC* caused by the vibration is expressed as

(43) $$DC=\frac{t_{2}-t_{1}}{T}$$

where $t_{1}=\left\{t|\frac{\partial \left(Y_{t}\right)}{\partial t}\geq 0,0<t<T\right\}$ and $t_{2}=\left\{t|\frac{\partial \left(Y_{t}\right)}{\partial t}\leq 0,t_{1}<t<t_{1}+T\right\}$, *Y_t_* is the vibration mode of the tool along *Y* direction, *t*_1_ is the time when the cutter cuts into the workpiece, *t*_2_ is the time when the cutter cuts out the workpiece, and *T* is the vibration period in *Y* direction.

The stress situation of cutting tools is divided into two situations to discuss:

When $nT\leq t\leq t_{1}+nT\left(n=1,2,3,\cdot \cdot \cdot \right)$, the rake face and the flank face of the tool are stressed at the same time.

When $t_{1}+nT\leq t\leq \;\left(n+1\right)T\left(n=1,2,3,\cdot \cdot \cdot \right)$, only the flank of the cutter is stressed.

## 3. Experiment and Validation

### 3.1. Setting Cutting Parameters

The workpiece material used in the experiment was RSA6061, which is a fine-grain aluminum alloy with an average grain size of 2–3 μm. The mechanical parameters of RSA6061 aluminum alloy are shown in Table 1. Such arrangement was to avoid the influence of large-grain 6061 aluminum alloy on the vibrations due to the grain boundaries and hard inclusions. The mechanical data in Table 1 are measured by a biomomentum dynamic mechanical test and an analysis system. Considering the common cutting parameters of the used ultra-precision lathe, the feed rate should be within the range of 0.5–10 μm, and the cutting depth should be within the range of 2–20 μm. The speed range used for the cutting experiments of micro-arc and large-arc tools was 500–2000 r/min. The diamond tool tip radii used for the experiments in this paper were: 50 μm, 200 μm, 400 μm, 800 μm and 1200 μm. Through these variables, the variation law of the maximum and average value of dynamic cutting force is obtained.

### 3.2. Cutting Force Measurement and Measurement of Vibration Signals

In this paper, the KS943L was used to collect the vibration signal and the sampling frequency is set to 40,000 Hz. The Kislter dynamometer 9119A-A1 was used to measure the cutting force and the sampling frequency is set to 200,000 Hz.

## 4. Results and Analysis

### 4.1. Validation of the Accuracy of the Measured Cutting Forces

**Figure 5:**
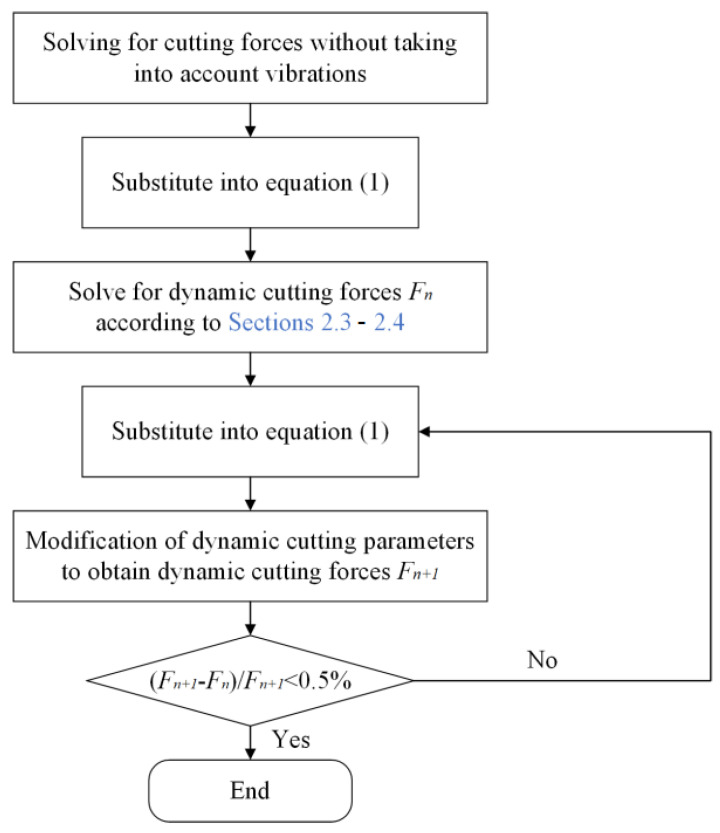
Flow chart for dynamic cutting force solution.

In this paper the dynamic cutting forces are solved, and the solution steps are shown in Figure 5.

**Figure 6:**
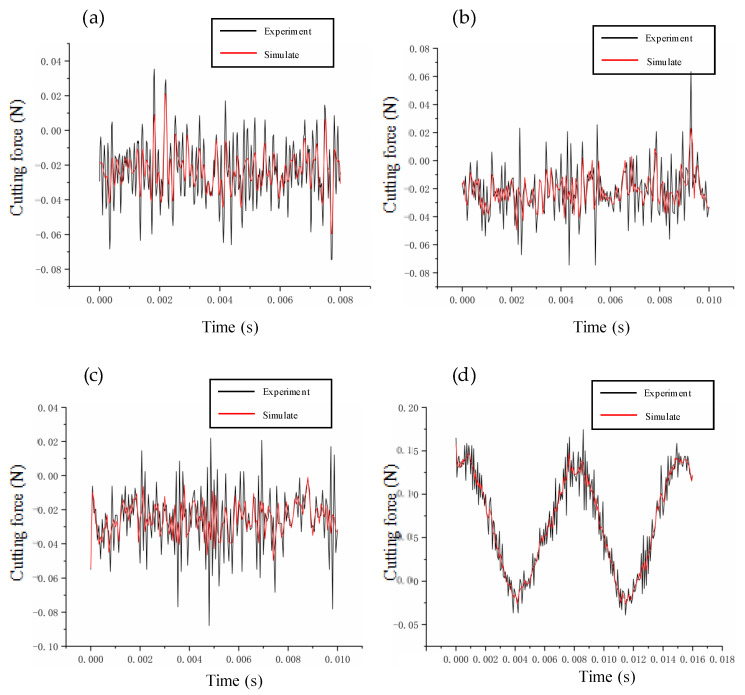
Verification of cutting forces at different depths of cut: (**a**) 2 μm; (**b**) 6 μm; (**c**) 8 μm; (**d**) 20 μm.

**Figure 7:**
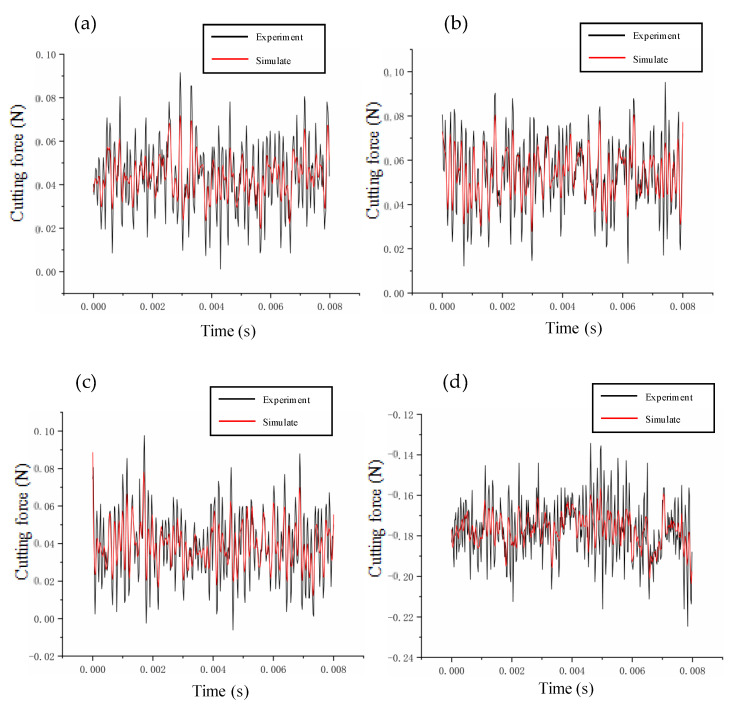
Verification of cutting forces at different feeds: (**a**) 0.5 μm/r; (**b**) 1 μm/r; (**c**) 3 μm/r; (**d**) 10 μm/r.

**Figure 8:**
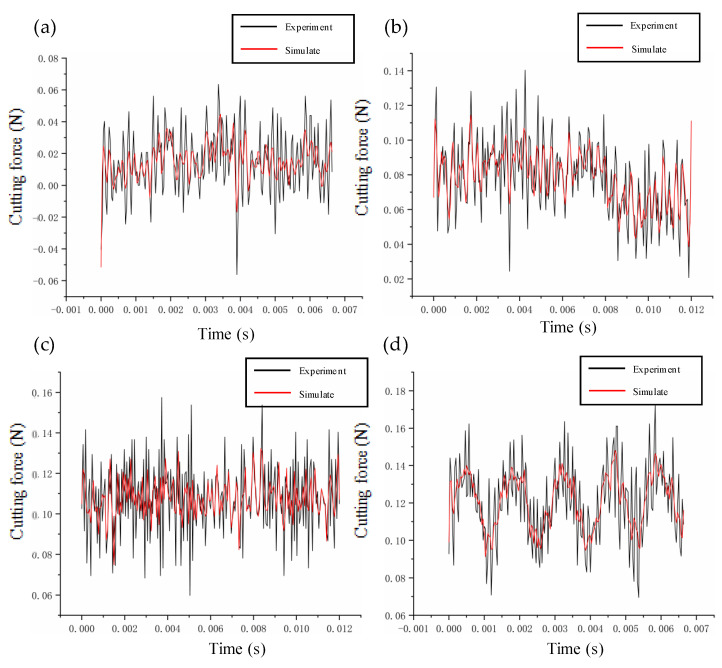
Verification of cutting forces at different rotation speeds: (**a**) 500 r/min; (**b**) 800 r/min; (**c**) 1200 r/min; (d) 2000 r/min.

In this paper, the prediction–correction method is adopted to model the dynamic cutting force. To verify the accuracy of the model, we compare the experimental and theoretical values of the dynamic cutting force for different cutting parameters. The depth of cut ranges from 2 μm to 20 μm, the feed rate ranges from 0.5 μm/r to 10 μm/r, and the speed ranges from 500 r/min to 2000 r/min. The verification results are summarized in Figure 6, Figure 7 and Figure 8. The theoretical modeling and actual measured cutting forces have a good accuracy and are in agreement in predicting the mechanism of the effect of cutting parameters on cutting forces.

### 4.2. Influence of Different Cutting Parameters and Tool Parameters (Radius of the Tool Tip Arc) on Cutting Forces

This paper differs from previous studies as it examines the effect of workpiece shape (radius size and surface equation) and diamond tool parameters on dynamic cutting force. To accurately evaluate the impact of various parameters on cutting forces, we analyze both the average cutting force and the maximum cutting force separately.

#### 4.2.1. Law of Influence of Cutting Parameters on Dynamic Cutting Forces

**Figure 9:**
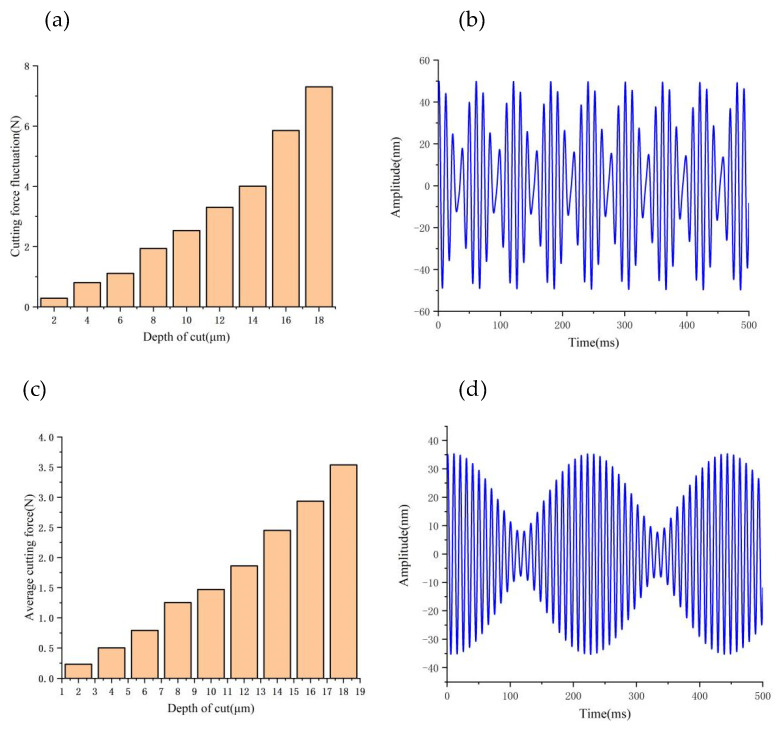
Effect of depth of cut on cutting forces and vibrations: (**a**) Variation of cutting force fluctuations values with depth of cut; (**b**) Tool tip vibration at 8 μm; (**c**) Variation of cutting force average values with depth of cut; (**d**) Tool tip vibration at 4 μm.

**Figure 10:**
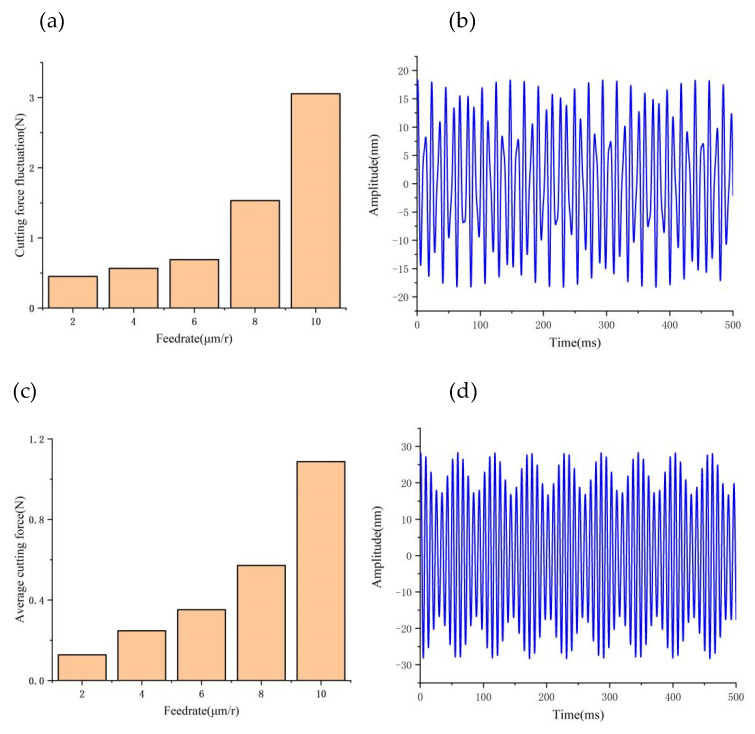
Effect of feedrate on cutting forces and vibrations: (**a**) Variation of cutting force fluctuations values with feed rate; (**b**) Tool tip vibration at 2 μm/r; (**c**) Variation of cutting force average values with feed rate; (**d**) Tool tip vibration at 6 μm/r.

**Figure 11:**
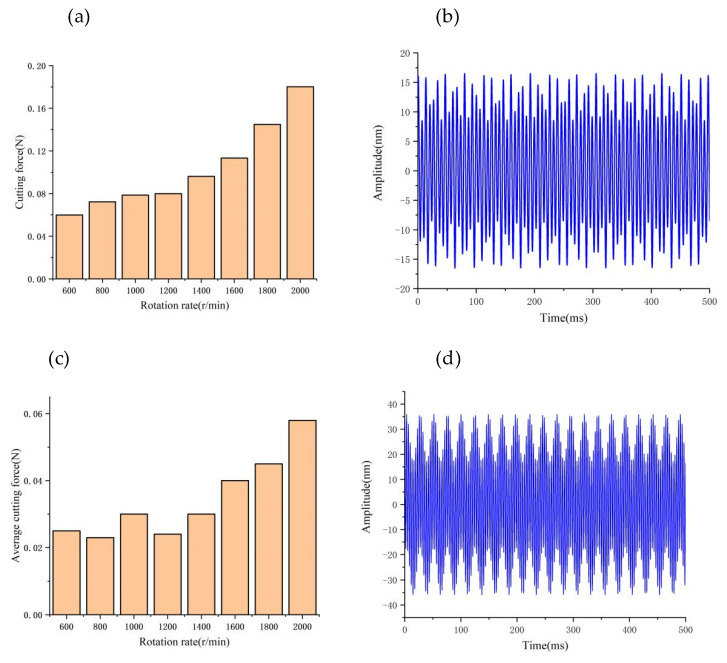
Effect of speed on cutting forces and vibrations: (**a**) Variation of cutting force fluctuations values with speed; (**b**) Tool tip vibration at 500 r/min; (**c**) Variation of cutting force average values with speed; (**d**) Tool tip vibration at 1200 r/min.

Figure 9, Figure 10 and Figure 11 provide a summary of the mean and maximum dynamic cutting forces at various depths of cut, feed rates spindle speeds. Unless otherwise indicated, this paper focuses on analyzing the cutting force component *F_z_*.

To maintain the consistency of other cutting parameters, we kept them unchanged. As the cutting depth increases, both the maximum and average values of dynamic cutting force also increase; however, it is noticeable that the average value of cutting force does not increase significantly, while the maximum value of cutting force increases rapidly. This phenomenon can be explained by analyzing the tool–workpiece vibration, as demonstrated in Figure 9c,d, where we observe an increase in the relative amplitude of the tool–workpiece vibration as the depth of cut increases. Based on the dynamic cutting force model, the increase in the relative amplitude of the tool–workpiece (from 35 nm to 50 nm) amplifies the range of the depth of cut changes, leading to large fluctuations in dynamic cutting force.

By keeping other cutting parameters unchanged, Figure 10a,b shows the influence of the feed rate on the dynamic cutting force, which increases as the feed rate increases. The increase in cutting width and actual cutting area as the feed rate increases results in an increase in the actual dynamic cutting forces. In Figure 10c,d, increasing the feed rate from 2 μm/r to 6 μm/r results in a slow increase in the relative vibration frequency and amplitude of tool–workpiece (from 18 nm to 27 nm). As a result, there is a slow but steady increase in the maximum dynamic cutting force.

By keeping the other cutting parameters unchanged and only varying the spindle speed, we found that the average value of dynamic cutting forces does not increase significantly with increasing rotating speed (as illustrated in Figure 11a,b); however, the fluctuation of dynamic cutting forces increases with increasing speed. By analyzing the relative vibration state between the tool and workpiece (as demonstrated in Figure 11c,d), we observed that the relative vibration amplitude between the tool and workpiece increases sharply (from 15 nm to 35 nm) with the increase in spindle speed. This phenomenon leads to a drastic fluctuation of dynamic cutting force between the tool and workpiece.

After discussing the influence of the aforementioned cutting parameters on dynamic cutting force, it can be concluded that as the cutting parameters increase, both the average and maximum values of dynamic cutting force gradually increase. However, due to the influence of relative vibration between the tool and workpiece, the range of fluctuation for the maximum dynamic cutting force value is larger. Consequently, when selecting cutting parameters, efforts should be made to minimize the fluctuation of the dynamic cutting force.

#### 4.2.2. Influence of the Radius of the Tool Tip on Dynamic Cutting Forces

**Figure 12:**
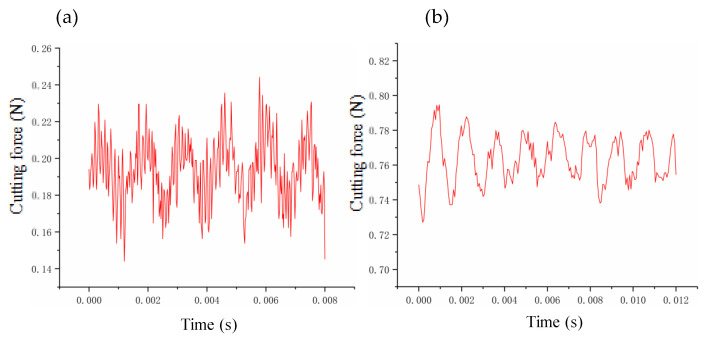
Influence of the radius of the tool tip arc on the cutting force (2 μm/r): (**a**) 50 μm; (**b**) 500 μm.

To improve the suppression of dynamic cutting force, we investigated the effects of tool tip arc radius, workpiece shape, and interpolation algorithm on the dynamic cutting force. Figure 12 illustrates the variation of average and maximum cutting forces at different tool tip radii. When keeping the same measuring area, workpiece shape, and size, we observed that the average and maximum cutting force values gradually increased with increasing tool tip radius, particularly at a larger feed rate of 2 μm/r. As discussed in Section 2.4, during large feed rates, the actual cutting area of the tool increases with the radius of the tip arc. Consequently, the actual cutting force (*F_y_*) also increases with the radius of the tip arc.

**Figure 13:**
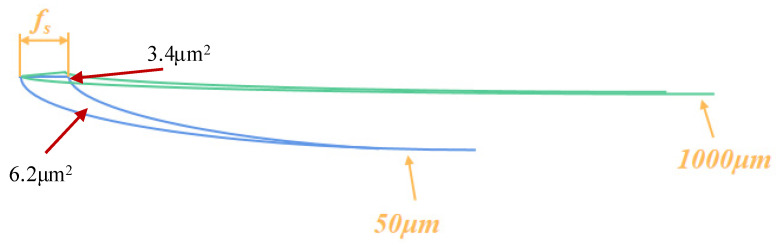
The actual cutting area at different tool nose radii (*f_s_* = 0.5 μm/r).

**Figure 14:**
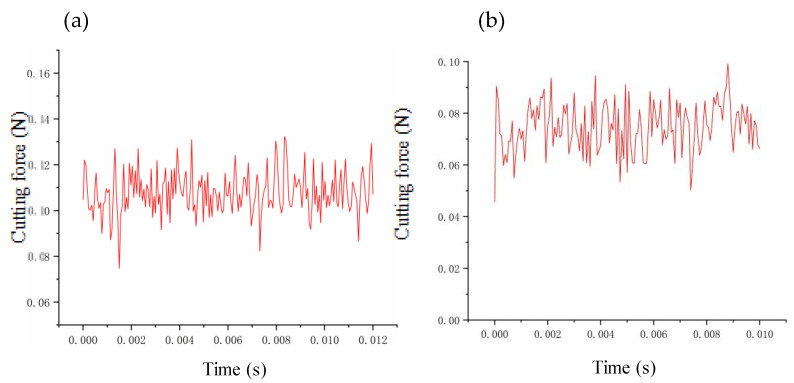
The actual cutting area at different tool nose radii (*f_s_* = 0.5 μm/r).

At a lower feed rate (0.5 μm/r), the cutting force component *F_y_* of the micro-arc tool (50 μm) is larger than that of the large-tip arc tool (500 μm). According to Equation (27), the chip thinning effect occurs when the large arc tool is fed at a very low speed (As shown in Figure 13, it can be clearly seen that the actual cutting area of micro-arc diamond tool is larger than that of large-arc diamond tool under very small feed.), so the actual cutting area of the rake face of the larger-arc diamond tool is smaller than that of the micro-arc diamond tool. Finally, the actual cutting force component *F_y_* of the large-arc diamond tool is smaller than that of the micro-arc diamond tool (Figure 14). Through calculation, we can get that when the feed rate is 1.25 μm/r; the cutting force component *F_y_* has nothing to do with the arc radius of the tool tip.

#### 4.2.3. Influence of Workpiece Shape on Dynamic Cutting Forces

**Figure 15:**
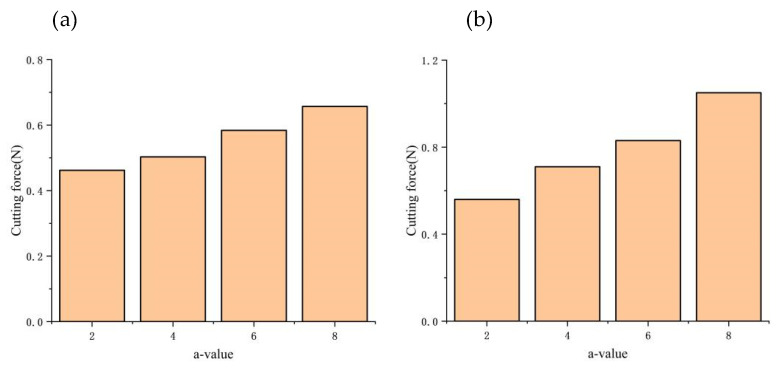
Effect of aspheric form on dynamic cutting forces: (**a**) Variation in average cutting forces; (**b**) Variation in cutting force fluctuation.

In this research paper, we investigate the impact of the aspheric radial equation on dynamic cutting forces using a parabolic surface with the radial equation *z* = *ax*^2^ (*a* > 0) as an example. Keeping the same vector height, we observe that the higher the value of a, the steeper the surface slope. The trend of dynamic cutting forces with the aspheric equation is presented in Figure 15. It is evident that with an increase in the slope of the cutting surface, the cutting force also increases. This phenomenon can be explained by Equation (30), and it is observed that the increase in the slope of the workpiece surface leads to an increase in cutting depth and actual feed rate. This increase further amplifies the dynamic cutting force. Additionally, the change in the dynamic cutting force affects the relative vibration between the tool and the workpiece and increases the fluctuation range of the dynamic cutting force.

**Figure 16:**
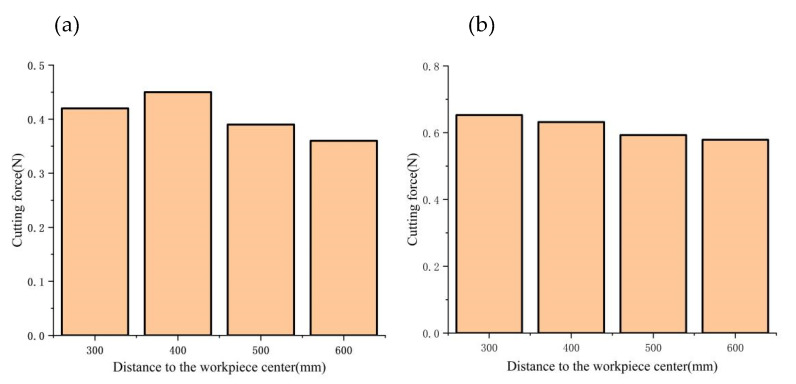
Effect of spheric form on dynamic cutting forces: (**a**) Variation in average cutting forces; (**b**) Variation in cutting force fluctuation.

In spherical machining, as illustrated in Figure 16, the average and maximum cutting force components decrease gradually with an increase in the spherical radius. Furthermore, as the radius of the machined spherical surface increases, the surface slope increases at the same vector height. The analysis of the relative vibration between the tool and workpiece indicates that the amplitude of the relative vibration increases with an increase in the slope of the cutting workpiece. Based on the conclusions drawn in Section 2, it can be inferred that higher relative vibration amplitudes lead to greater fluctuations in dynamic cutting forces.

**Figure 17:**
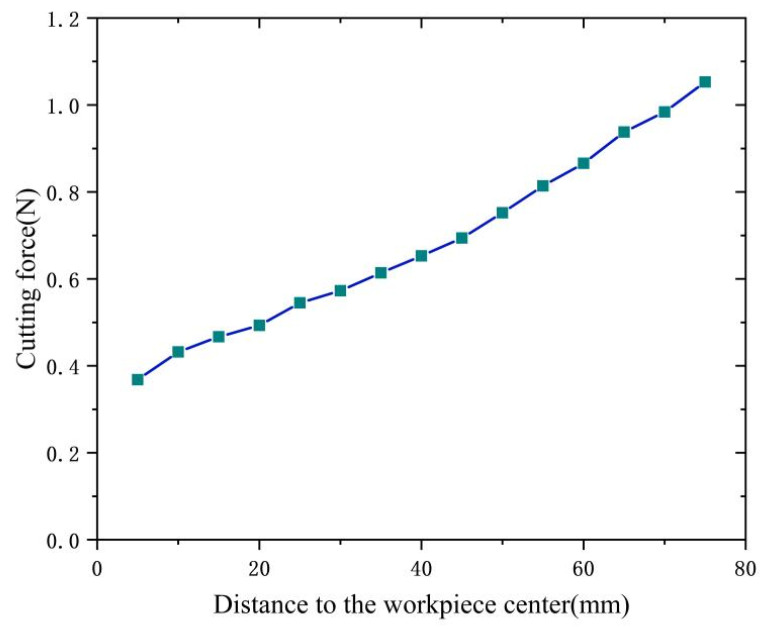
Influence of the radial dimension of the workpiece on the dynamic cutting forces.

In this paper, we investigate the impact of the distance between the tool and the workpiece center on the dynamic cutting force. As illustrated in Figure 17, the mean and maximum values of dynamic cutting forces augment as the radial dimension of the workpiece increases. On the one hand, the increase of workpiece radius leads to a rise in the cutting amount per unit time with a corresponding increase in cutting speed, which ultimately results in a higher cutting force. On the other hand, as portrayed in Table 2, the cutting vibration frequency and amplitude progressively amplify as the workpiece radius increases. From a vibration perspective, a larger vibration amplitude contributes to a greater fluctuation of dynamic cutting force.

In Table 2, *r_n_* denotes the distance from the cutting position to the center on the workpiece surface.

### 4.3. Effect of Different Interpolation Algorithms on Dynamic Cutting Forces

The interpolation algorithm used plays a crucial role in achieving the desired depth of cut during the machining of aspheric surfaces, given the model described in Section 2.2. Additionally, selecting an appropriate interpolation algorithm is paramount in minimizing surface roughness resulting from the machining process.

**Figure 18:**
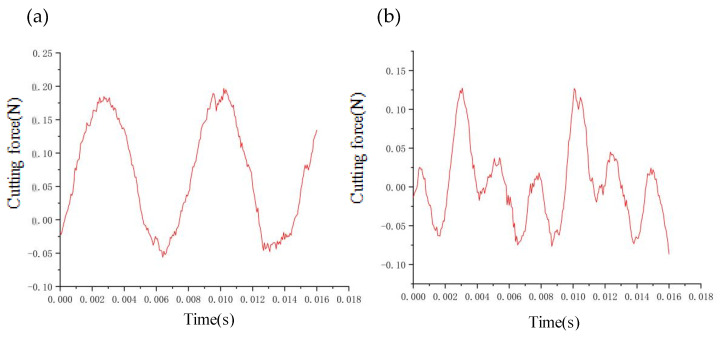
Effect of different interpolation algorithms on dynamic cutting forces: (**a**) Equal feed interpolation algorithm; (**b**) Equal residual height interpolation algorithm.

From Figure 18, it is evident that when the slope of the machining region is steep, the equal feed interpolation algorithm produces higher average and maximum values of dynamic cutting force compared to the equal residual height interpolation algorithm. The underlying principle of these two interpolation algorithms suggests that the cutting depth of the equal feed interpolation algorithm increases as the slope of the workpiece surface increases. This explains why the average dynamic cutting force of the equal feed interpolation algorithm exceeds that of the equal residual height interpolation algorithm.

**Figure 19:**
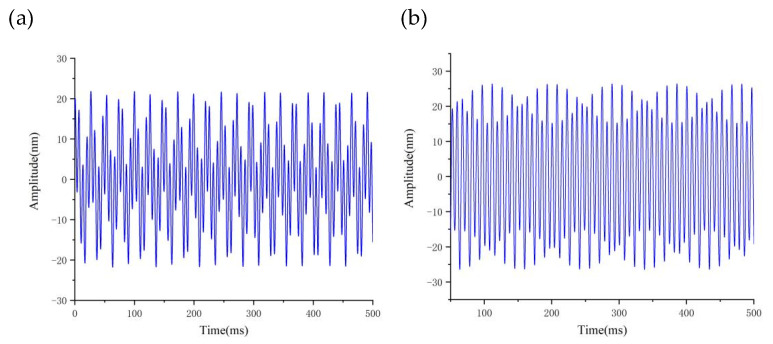
Effect of different interpolation algorithms on dynamic cutting forces: (**a**) Equal feed interpolation algorithm; (**b**) Equal residual height interpolation algorithm.

By measuring the relative vibration between tool and workpiece of the two interpolation algorithms (Figure 19), it can be seen that the amplitude of the interpolation algorithm with equal residual height (18 nm) is smaller than that of the interpolation algorithm with equal feed (25 nm) in the higher slope region. This explains why the maximum dynamic cutting force of the interpolation algorithm with equal residual height is smaller than that of the interpolation algorithm with equal feed rate.

The analysis above demonstrates that selecting an appropriate interpolation algorithm can efficiently mitigate dynamic cutting forces and reduce roughness values on the workpiece surface. In the upcoming research, the authors aim to develop practical algorithms that effectively suppress the fluctuation of dynamic cutting forces by delving into the mechanism of dynamic cutting force variation.

## 5. Interpolation Point Optimization Algorithm for Reducing Dynamic Cutting Force

**Figure 20:**
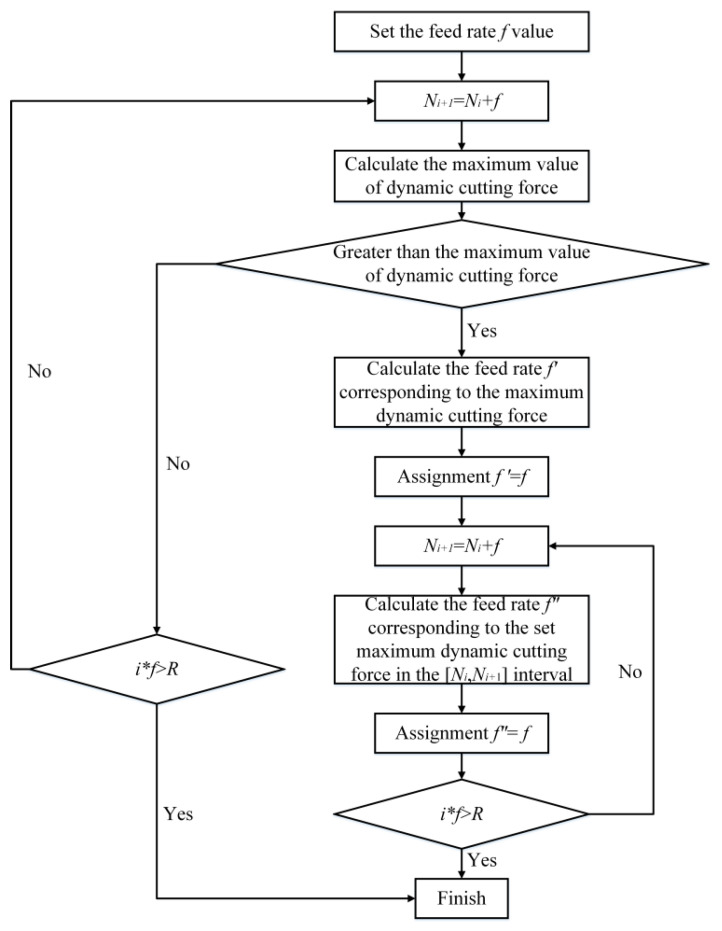
Flow chart of interpolation point position optimization.

In an effort to minimize the impact of dynamic cutting force on optical surface reflectivity, the interpolation point position is deduced based on the previously determined maximum value of dynamic cutting force. The specific calculation process is illustrated in Figure 20, with the maximum value of dynamic cutting force being adjustable contingent upon the actual machining parameters. In this study, the input sample denotes the position of the preceding interpolation point, whereas the output value pertains to the position of the current interpolation point.

The specific steps of the algorithm are as follows:

- Firstly, the initial feed rate value *f* is set, and the *N* regions are divided along the meridian with the initial feed value, and whether the maximum dynamic cutting force in each region exceeds the set value is calculated in sequence.
- If it does not exceed the set value, continue to plan the interpolation point according to the initial value of the feed.
- If it exceeds the set value, calculate the limit cutting depth and the corresponding feed rate in this interval, and redivide the remaining area with this feed rate.
- In the next area, continue to calculate the limit cutting depth and feed rate, and continue to divide the remaining areas with this feed rate.
- Repeat the above steps until the meridian planning of the workpiece is completed.

By leveraging the aforementioned optimization algorithm, the interpolation point position can be determined, and the dynamic cutting force can be acquired during the actual machining process, as depicted in Table 3 and Table 4. Irrespective of the surface shape (spherical or aspherical), the interpolation point position optimization algorithm leads to a noticeable and consistent reduction of dynamic cutting force under varying machining parameters, ranging from 0.1 to 0.6 N. This experimental outcome attests to the reliability and effectiveness of the optimization algorithm.

## 6. Conclusions

This study presents a dynamic cutting force model for both spherical and aspherical machining processes. The study takes into account the impact of cutting depth, feed rate, and spindle speed on the average and maximum values of dynamic cutting force, as well as the effect of tool tip arc radius, workpiece shape, and interpolation algorithm, which is the first of its kind. Based on the analytical model and experimental results, the following conclusions can be drawn from this study.

1. This study models the actual chip thickness, actual cutting width, and actual shear angle for cases of cutting vibrations. Tool–workpiece contact is separately discussed under different vibration conditions, and a real-time dynamic cutting force model is developed for the tool under these conditions. Additionally, a model of the actual depth of cut and feed rate is developed, considering different interpolation algorithms, and a novel dynamic cutting force model is developed for equal feed interpolation algorithms and equal residual height interpolation algorithms.
2. Based on the experimental results, it can be concluded that the theoretical model provides a good estimation of the average dynamic cutting force and can also predict the fluctuation range of the dynamic cutting force with a relative error of approximately 10%. Through this theoretical model and experimental analysis, an understanding of the varied behavior of the maximum and average dynamic cutting force with respective cutting parameters is obtained, furnishing a basis for further optimization of cutting and tool parameters. Moreover, it is worth noting that the model developed in this study can be applied to plastic materials of various compositions.
3. In this study, we investigate the impact of the arc radius of a diamond tool tip and the interpolation algorithm on dynamic cutting force for the first time. At smaller feed rates (0.5 μm/r), we observe that the cutting force components for small-arc tools are greater than those for large-arc tools. This phenomenon can be attributed to chip thinning, which reduces the actual cutting area of large circular arc diamond tools. Therefore, by selecting the appropriate tool tip arc radius for different feed rates, the dynamic cutting force can be effectively reduced.

Furthermore, we measure the relative vibration between the tool and the workpiece using two interpolation algorithms. We find that the vibration mode of the interpolation algorithm with equal residual height is smaller than that of the interpolation algorithm with equal feed in the area with a large slope. This results in a smaller fluctuation range of dynamic cutting force for the equal residual height interpolation algorithm compared to the equal feed interpolation algorithm.

4. In this paper, a new algorithm for planning interpolation points is utilized to optimize their position during the machining process. The experimental results demonstrate that this optimization algorithm can effectively reduce the dynamic cutting force, thus validating its reliability and practicality. According to the interpolation algorithm, the initial feed rate of 1–2 μm/r can effectively reduce the dynamic cutting force and enhance machining efficiency. To ensure optimal machining efficiency, a spindle speed of 1000–1200 r/min is recommended. Additionally, it should be noted that the dynamic cutting force decreases as the cutting depth value decreases.

## Figures and Tables

**Table 1:** Mechanical Properties of RSA 6061 Aluminum Alloy.

Hardness (GPa)	Modulus of Elasticity (GPa)	Stress Coefficient	Yield Strength (GPa)
1	70	3.5	300

**Table 2:** Variation of vibration frequency and amplitude with equal-feed interpolation algorithm.

*r_n_* (mm)	Frequency (Hz)/Amplitude(nm)									
5	135/2.3	273/6.2	794.4/ 8.0		1050/3.2	1200/2.5		2200/4.5	4536/3.4	6750/2.0
10	135/2.4	273.4/7.6	795/ 12.5		1050/4.5	1200/4.8		2200/6.8	4524.7/4.8	6750/3.8
15	135/6.7	273.6/8.4	795.4/ 10.9		1050/10.5	1139/6.7	1245/5.4	2200/9.7	4526/4.7	6750/3.9
40	135/7.6	275.5/8.5	792.5/14.9		1050/14.8	1047/15.8	1391/6.7	2200/10.8	4517/5.9	6750/3.8
45	135/7.9	275.8/8.0	757.3/15.2	946.3/7.2	1050/18.7	1023/21.9	1423/8.9	2200/13.8	4533/6.8	6750/4.4
75	135/8.9	277.9/9.7	558.6/18.7	1104.7/8.5	1050/22.8	874/30.9	1640/9.2	2200/14.9	4568/7.9	6750/4.8

**Table 3:** Comparison of dynamic cutting force before and after using optimization algorithm (spherical surface).

Feed Rate (μm/r)	Cutting Depth (μm)	Conventional Cutting (N)	Optimization Algorithm (N)	Declining Quantity (N)
0.5	1	0.35	0.24	0.11
	2	0.42	0.30	0.12
	5	0.51	0.34	0.17
	10	0.59	0.39	0.20
	20	0.64	0.45	0.19
1	1	0.34	0.21	0.13
	2	0.49	0.38	0.11
	5	0.52	0.41	0.11
	10	0.65	0.51	0.14
	20	0.96	0.79	0.17
2	1	0.42	0.30	0.12
	2	0.49	0.34	0.15
	5	0.59	0.40	0.19
	10	0.72	0.59	0.13
	20	1.29	0.90	0.39
4	1	0.62	0.43	0.19
	2	0.79	0.59	0.20
	5	0.91	0.64	0.27
	10	1.04	0.79	0.25
	20	1.45	1.10	0.35
6	1	0.78	0.52	0.26
	2	0.89	0.64	0.25
	5	1.15	0.84	0.31
	10	1.46	1.05	0.41
	20	1.92	1.35	0.57

**Table 4:** Comparison of dynamic cutting force before and after using optimization algorithm (aspherical surface).

Feed Rate (μm/r)	Cutting Depth (μm)	Conventional Cutting (N)	Optimization Algorithm (N)	Declining Quantity (N)
0.5	1	0.49	0.36	0.13
	2	0.56	0.42	0.14
	5	0.65	0.46	0.19
	10	0.73	0.51	0.22
	20	0.78	0.57	0.21
1	1	0.48	0.33	0.15
	2	0.63	0.5	0.13
	5	0.66	0.53	0.13
	10	0.79	0.63	0.16
	20	1.1	0.91	0.19
2	1	0.56	0.42	0.14
	2	0.63	0.46	0.17
	5	0.73	0.52	0.21
	10	0.86	0.71	0.15
	20	1.43	1.02	0.41
4	1	0.76	0.55	0.21
	2	0.93	0.71	0.22
	5	1.05	0.76	0.29
	10	1.18	0.91	0.27
	20	1.59	1.22	0.37
6	1	0.92	0.64	0.28
	2	1.03	0.76	0.27
	5	1.29	0.96	0.33
	10	1.6	1.17	0.43
	20	2.06	1.47	0.59

## Data Availability

Data underlying the results presented in this paper are not publicly available at this time but may be obtained from the authors upon reasonable request.
